# Supplementary material for: Seventy-Two-Hour LRRK2 Kinase Activity Inhibition Increases Lysosomal GBA Expression in H4, a Human Neuroglioma Cell Line
Source: Int J Mol Sci. 2022 Jun 22;23(13):6935. doi: 10.3390/ijms23136935 (PMC9266636; doi:10.3390/ijms23136935)
Supplement: Supplementary file 1 [file ijms-23-06935-s001.zip › ijms-1761077-supplementary.pdf]

International Journal of
Molecular Sciences

Article

Seventy-Two-Hour LRRK2 Kinase Activity Inhibition Increases Lysosomal GBA Expression in H4, a Human Neuroglioma Cell Line

Clara Ruz [1,2], José Luis Alcantud [2], Francisco Vives [1,2], Francisco Arrebola [2,3], John Hardy [4], Patrick A. Lewis [4,5], Claudia Manzoni [6] and Raquel Duran [1,2]*

[1] Department of Physiology, Faculty of Medicine, Universidad de Granada,18016, Granada, Spain; clararuz@ugr.es (C.R.); fvives@ugr.es (F.V.)
[2] Institute of Neurosciences "Federico Olóriz", Centro de Investigación Biomédica (CIBM), Universidad de Granada, 18016 Granada, Spain; clararuz@ugr.es (CR); jlalcantud@ugr.es (JLA); fav@ugr.es (FA)
[3] Department of Histology, Faculty of Medicine, Universidad de Granada, 18016 Granada, Spain; fav@ugr.es
[4] Department of Neurodegenerative Disease, UCL Queen Square Institute of Neurology, WC1N 3BG London, UK; j.hardy@ucl.ac.uk (J.H.); plewis@rvc.ac.uk (P.A.L.)
[5] Department of Comparative Biomedical Science, Royal Veterinary College, Royal College Street, NW1 0TU London, UK
[6] Department of Pharmacology, UCL School of Pharmacy, WC1N 1AX London, UK; c.manzoni@ucl.ac.uk
* Correspondence: rduran@ugr.es

Supplementary Materials

Citation: Ruz, C.; Alcantud, J.L.; Vives, F.; Arrebola, F.; Hardy, J.; Lewis, P.A.; Manzoni, C.; Duran, R. Seventy-Two Hour LRRK2 Kinase Activity Inhibition Increases Lysosomal GBA Expression in H4, a Human Neuroglioma Cell Line. *Int. J. Mol. Sci.* **2022**, *23*, 6935. https://doi.org/10.3390/ijms23136935

Academic Editor: Alessandro Cannavo

Received: 23 May 2022
Accepted: 20 June 2022
Published: 22 June 2022

Publisher's Note: MDPI stays neutral with regard to jurisdictional claims in published maps and institutional affiliations.

Copyright: © 2022 by the authors. Licensee MDPI, Basel, Switzerland. This article is an open access article distributed under the terms and conditions of the Creative Commons Attribution (CC BY) license (https://creativecommons.org/licenses/by/4.0/).

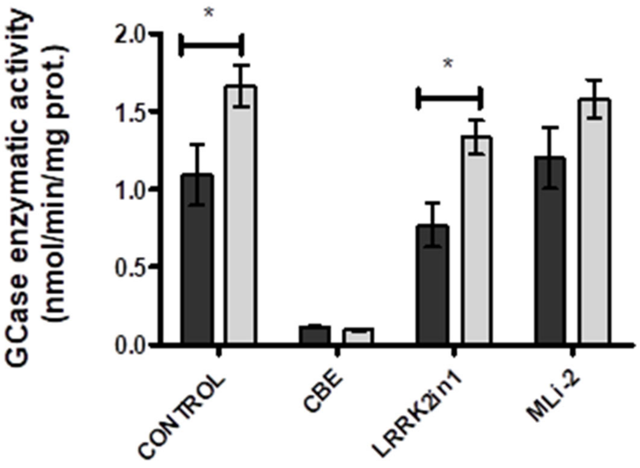

Figure S1. GCase enzymatic activity of cell lysates treated with DMSO (as control), LRRK2in1, MLi2 and CBE after 24 h (black bars) and 72 h (grey bars) incubations.

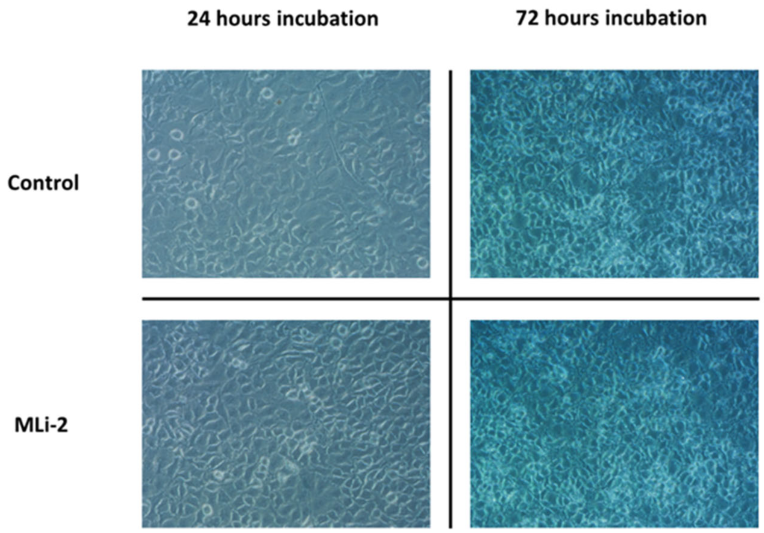

Figure S2. Representative images (10X) from inverted phase-contrast microscope of H4 human neuroglioma cells after incubation with MLi-2 (600nM) or DMSO (0.15% v/v) as control for 24 and 72h.
